# Molecular Dissection of DAAM Function during Axon Growth in Drosophila Embryonic Neurons

**DOI:** 10.3390/cells11091487

**Published:** 2022-04-28

**Authors:** István Földi, Krisztina Tóth, Rita Gombos, Péter Gaszler, Péter Görög, Ioannis Zygouras, Beáta Bugyi, József Mihály

**Affiliations:** 1 Biological Research Centre, Institute of Genetics, Eötvös Loránd Research Network, Temesvári krt. 62, H-6726 Szeged, Hungary; foldi.istvan@brc.hu (I.F.); toth.krisztina@brc.hu (K.T.); gombos.rita@brc.hu (R.G.); gorog.peter@brc.hu (P.G.); izygouras@bio.auth.gr (I.Z.); 2 Doctoral School of Multidisciplinary Medical Science, Faculty of Medicine, University of Szeged, H-6725 Szeged, Hungary; 3 Department of Biophysics, Medical School, University of Pécs, Szigeti str. 12, H-7624 Pécs, Hungary; peter.gaszler@aok.pte.hu (P.G.); beata.bugyi@aok.pte.hu (B.B.); 4 Department of Genetics, University of Szeged, H-6726 Szeged, Hungary

**Keywords:** cytoskeleton, formin, axon, nervous system, development, Drosophila

## Abstract

Axonal growth is mediated by coordinated changes of the actin and microtubule (MT) cytoskeleton. Ample evidence suggests that members of the formin protein family are involved in the coordination of these cytoskeletal rearrangements, but the molecular mechanisms of the formin-dependent actin–microtubule crosstalk remains largely elusive. Of the six *Drosophila* formins, DAAM was shown to play a pivotal role during axonal growth in all stages of nervous system development, while FRL was implicated in axonal development in the adult brain. Here, we aimed to investigate the potentially redundant function of these two formins, and we attempted to clarify which molecular activities are important for axonal growth. We used a combination of genetic analyses, cellular assays and biochemical approaches to demonstrate that the actin-processing activity of DAAM is indispensable for axonal growth in every developmental condition. In addition, we identified a novel MT-binding motif within the FH2 domain of DAAM, which is required for proper growth and guidance of the mushroom body axons, while being dispensable during embryonic axon development. Together, these data suggest that DAAM is the predominant formin during axonal growth in *Drosophila*, and highlight the contribution of multiple formin-mediated mechanisms in cytoskeleton coordination during axonal growth.

## 1. Introduction

Recent advances in the field of neuroscience revealed the fine structure of the axonal cytoskeleton and further demonstrated the importance of actin and microtubule (MT) interactions during axonal development [1,2]. Axonal growth cones (GCs) are particularly rich in both cytoskeletal elements, and movement of the axons is governed by cytoskeleton rearrangements induced by guidance molecules and adhesion proteins [3]. Several modes of actin–microtubule crosstalk have been described which may be involved in axonal growth [4]. For example, microtubules defasciculating from the central bundle make contact with the actin arcs of the transition zone of the GC, while the MTs reaching out to the peripheral zone can be captured by F-actin bundles and extend preferentially alongside filopodial actin, and also the assembly of branched actin arrays is possible from microtubule plus ends [5,6,7]. Most of these interactions are mediated by cytolinkers, proteins that are able to make contact with both cytoskeletal elements. Although a growing number of proteins, such as spectraplakins (ACF7/MACF1 in mammals, Shot in Drosophila), Drebrin, Gas2-like 1, NAV1, XMAP215 and formins, are clearly implicated in linking of the regulation of actin and MT dynamics in growth cones, many aspects of their mechanisms remained largely unexplored.

Formins are well known to play an essential role in the formation of unbranched actin filaments; however, an increasing body of work has suggested that they can also regulate the microtubule cytoskeleton. The most conserved structural elements of all formins are the two formin homology domains (FH1 and FH2) [8]. The FH1 domain contains polyproline motifs, which serve as a binding surface for profilin-bound actin monomers, whereas the FH2 is the main functional domain having a role in actin nucleation and filament elongation [9,10]. Members of the diaphanous-related formin (DRF) subfamily, such as Dia, DAAM and FRL, contain other conserved domains as well, including a GTPase-binding domain (GBD), a diaphanous inhibitory domain (DID) and a diaphanous autoregulatory domain (DAD). Interaction between the DID and DAD domains provides an autoinhibited conformation, whereas binding of a small GTPase (such as Rho, Rac or cdc42) to the GBD destabilizes the DID–DAD interaction which in turn partially activates the DRFs [11,12]. In addition to actin binding, several studies showed that formins can directly interact with microtubules and they play an important role in the formation of stable MTs [13,14,15,16,17,18,19,20]. These data suggested that in vitro and in simple cellular assays, most formins exhibit an MT stabilization activity, which is usually dependent on their FH2 domain [21], although some formins contain other MT-binding domains [15,16]. In addition to these domains, few formins were shown to be able to bind MTs via their positively charged C-terminal tail (CT) regions [19,22,23], albeit binding strength seems to be lower than for the FH2 domain [18]. Importantly, it has also been revealed that the actin assembly activity of formins is not necessary for the induction of MT stabilization nor for direct MT interaction [17,19,22]. Collectively, these observations suggested that the main MT-binding surface of formins resides in the FH2 domain. Interestingly, however, the FH2 domain is also implicated in another type of MT interaction, attained by binding to MT plus-end tracking proteins (+TIPs), such as end-binding protein 1 (EB1), adenomatous polyposis coli (APC) or cytoplasmic linker protein 170 (CLIP-170) [14,17,24]. The concerted action of formins and +TIPs is likely to represent another important mechanism of actin and MT coordination in neurons, and possibly in other cell types [14,24,25,26]. Thus, formins can be coupled to MTs in several distinct ways that might involve direct MT-binding and/or interactions with MT-binding proteins, such as +TIPs. Whereas these are not necessarily mutually exclusive alternatives, it remained an important question to understand the functional impact of these mechanisms in a consistent in vivo model system, while keeping in mind that formins can also exert an indirect effect on MT regulation, via modulation of the actin cytoskeleton. 

In a previous study, we showed that the DAAM and FRL formins have a redundant contribution to development of the mushroom body (MB) neurons of the adult *Drosophila* brain [27]. We found that axonal growth of the Kenyon cells was significantly more compromised in *DAAM; flr* double mutants as compared to single mutant animals. To extend these studies, we investigated the possible redundant role of DAAM and FRL in development of the embryonic central nervous system (CNS) and axonal growth. We found that the *frl* null mutant embryos showed no developmental defects in the CNS, and our double-mutant analysis revealed no indications for redundancy between DAAM and FRL in the embryonic nervous system, suggesting that of these two formins, only DAAM is crucial for embryonic axonal growth. As to the mechanisms of DAAM, we have formerly shown that the growth speed of MTs is reduced and organization of the MT cytoskeleton is altered in *DAAM* mutant primary neurons [22]. We have also shown that Drosophila DAAM can directly interact with taxol-stabilized MTs via its FH2 domain and CT region, and through this direct interaction, DAAM stabilizes MTs against cold-induced depolymerization in vitro. Our results also revealed that a C-terminal fragment of DAAM (CDAAM) including the FH1, FH2, DAD and CT domains, can crosslink and co-align the F-actin and MT filaments. Together, these results strongly suggested that DAAM has a direct effect on MT dynamics during axonal growth. However, we have also noticed that the effect of an actin-destabilizing reagent (Latrunculin A) on MT growth was largely comparable to what we measured in *DAAM* mutant neurons. Therefore, how DAAM regulates the MT-cytoskeleton during axonal growth remained an unanswered question. 

In this paper, we identify DAAM as the sole formin member necessary during the development of the embryonic nervous system of *Drosophila*. To dissect the role of DAAM at a molecular level and to address how its MT side-binding and actin processing activities regulate the axonal cytoskeleton, we performed rescue experiments with wild-type and mutant forms. Our current and previous experiments demonstrate that the actin-processing activity of DAAM is indispensable for normal neural growth in every developmental aspect studied so far. In addition, we identified a novel MT-binding motif within the FH2 domain of DAAM, and we demonstrate that it plays a role in vivo during mushroom body development, while it appears dispensable during embryonic neuronal development.

## 2. Materials and Methods

### 2.1. Fly Stocks and Genetics

Flies were raised at 25 °C under standard conditions. The following mutant strains were used: *w^1118^* (BL #3605), *Elav-Gal4* (BL #8760 and 8765), OK107-Gal4 (BL #854) provided by the Bloomington *Drosophila* Stock center, *dDAAM^Ex4^* [28], *frl^59^* [29], *DAAM^Ex4^; frl^59^/TM3, twi-Gal4, UAS-EGFP, UAS-DAAM* [30], *UAS-DAAM^I732A^* [28], *UAS-DAAM^K881A^* [31], *UAS-DADG* [28] and *UAS-CDAAM* [30]. Where necessary, zygotic mutants were selected by using a *CyO, twi-Gal4, UAS-EGFP* or *TM3*, *twi-Gal4, UAS-EGFP* balancer chromosome. For live imaging, the *ElavGal4,EB1::GFP* line was crossed to *UAS-CDAAM* or *UAS-CDAAM^I732A^*. The *UAS-DAAM^FH2R/K-A^, UAS-CDAAM^I732A^* and *UAS-CDAAM^FH2R/K-A^* transgenic constructs were generated as described below. Drosophila transgenesis was carried out by using the PhiC31 integrase and all transgenes were integrated into the *attP40* landing site on the second chromosome.

### 2.2. Molecular Biology

For transgenesis, CDS of all DAAM constructs were cloned into the pWalium5moe vector, which is derived from pWalium10moe by the removal of a 5xUAS sequence. Point mutations were generated by site-directed mutagenesis by using standard protocols (for primers, see Table S1). For bacterial protein expression DAAM FH2, FH2^R/K-A^ constructs were cloned into pGex6p1 or pGex2T. For S2 cell protein expression, DAAM FH2, FH2^R/K-A^, CDAAM, CDAAM^I732A^, CDAAM^FH2R/K-A^ constructs were cloned into either pAFW or pAGW destination vectors by following a standard Gateway cloning procedure.

### 2.3. Protein Expression and GST Pull-Down

N-terminally GST-tagged DAAM FH2, FH2^R/K-A^ recombinant proteins were expressed and purified as described earlier [32]. For GST pull-down, recombinant proteins (5 µg of each) were immobilized on glutathione-sepharose 4B beads (GE Healthcare, Chicago, IL, USA) in storage buffer (50 mM Hepes, pH: 7.5; 5 mM DTT; 50 mM NaCl; 5% glycerol; 1% sucrose). Sepharose beads were then incubated for 1 h at room temperature (RT) with purified tubulin (Cytoskeleton, Denver, CO, USA), which was previously dissolved in PEM buffer (80 mM Pipes, pH: 7.0; 2 mM MgCl_2_; 0.5 mM EGTA) and then diluted in microtubule-binding buffer (MBB; 10 mM Hepes, pH: 7.0; 1 mM MgCl_2_; 1 mM EGTA; 1 mM DTT; 0.5 mM Thesit) to have a final concentration of tubulin at 0.5 µM. Beads were then washed in MBB and the proteins were eluted in SDS-PAGE sample buffer. Proteins were analyzed by SDS-PAGE and Western blot by using a standard procedure. GST-tagged proteins were detected by Coomassie blue staining and tubulin was detected by an anti-tubulin antibody (1:1000; DM1A, Merck KGaA, Darmstadt, Germany) in combination with HRP-conjugated anti-mouse IgG (1:10,000; Jackson ImmunoResearch Europe Ltd, Cambridgeshire, UK). 

### 2.4. MT Co-Sedimentation

MT co-sedimentation was performed as described by Fassier et al. in 2018 [33] with some modifications. S2 cells were transfected with either pAFW-DAAM-FH2 or pAFW-DAAM-FH2^R/K-A^ plasmids by using the Effectene transfection reagent (QIAGEN Sciences Inc., Germantown, MD, USA). After 24 h of culturing at 27 °C, the cells were harvested (1000× *g*, 2 min, 4 °C) and washed in PEM buffer supplemented with a protease inhibitor cocktail (cOmplete^TM^, F. Hoffmann-La Roche Ltd., Basel, Switzerland). Pellets were resuspended in PEM buffer and the cells were lysed by sonication. Total lysates were spun at 150,000× *g*, 10 min, 4 °C in an Optima MAX-XP benchtop ultracentrifuge (Beckman Coulter Inc., Brea, CA, USA) using TLA120.1 rotor. Supernatants were collected and supplemented with 20 µM Taxol (SigmaAldrich) and 1 µM GTP (SigmaAldrich). Samples were incubated at 37 °C for 30 min to let the MTs polymerize. The resulting extracts were layered onto a 10% sucrose cushion in PEM buffer (one third of the final volume). The samples were spun at 180,000× *g*, 15 min, 37 °C. Supernatants were collected in empty tubes and the pellets were resuspended in the original volume of PEM buffer. The proteins were analyzed by SDS-PAGE and Western blot. Equal volumes were loaded from the supernatant and pellet fractions. DAAM constructs were detected by a mouse anti-Flag antibody (1:500; SigmaAldrich) and tubulin was detected as described for GST pull-down.

### 2.5. SDS-PAGE and Western Blot Analysis of Embryonic Lysates

Stage 11 and 16–17 embryos were collected and homogenized in a lysis buffer (25 mM Tris-HCl, pH:7.5; 150 mM NaCl; 0.1% SDS; 0.5% Na-deoxycholate; 1% TritonX-100) supplemented with a protease inhibitor cocktail (cOmplete^TM^, F. Hoffmann-La Roche Ltd, Basel, Switzerland). Homogenates were spun at 12,000× *g*, 10 min, 4 °C and supernatants were analyzed by SDS-PAGE and Western blot by using a standard procedure. DAAM was detected by a rabbit anti-DAAM polyclonal antibody (1:500; [34]) in combination with HRP-conjugated anti-rabbit-IgG secondary antibody (1:10,000; Jackson Immunoresearch). FRL was detected with a rat anti-FRL polyclonal antibody (1:1000; unpublished data, described in Tóth et al., under revision in Cells) in combination with an HRP-conjugated anti-Rat-IgG (1:10,000; Jackson Immunoreserach). Rat anti-α-Actinin (1:10,000; Babraham Institute, Cambridge, UK) or rat anti-actin (1:10,000; Babraham Institute, Cambridge, UK) antibodies were applied to detect proteins that were used as loading controls.

### 2.6. Primary Neuronal Cultures and Immunohistochemistry

Primary neuronal cells were obtained from stage 11 Drosophila embryos as described previously by Sanchez-Soriano et al. in 2010 [35]. Primary neurons were cultured for 6 h or 24 h in vitro (HIV), and then fixed by following a procedure described by Xu et al. in 2013 [36]. Briefly, cells were pre-fixed for 1 min in a solution containing 0.3% (*w*/*v*) glutaraldehyde (Electron Microscopy Sciences, Hatfield, PA, USA) and 0.25% TritonX-100 in Cytoskeleton buffer (CB; 10 mM PIPES, pH 7; 150 mM NaCl; 5 mM EGTA; 5 m glucose and 5 mM MgCl_2_), then post-fixed for 15 min in 2% (*w*/*v*) glutaraldehyde in CB. After fixation, cells were permeabilized and blocked-in blocking buffer (0.2% *w*/*v* BSA, 0.3% Triton X-100 in CB) for 30 min. MT cytoskeleton was detected by anti-tubulin (1:1000; DM1A) antibody in combination with Alexa405-anti-mouse IgG (1:600; Thermo Fisher Scientific Inc., Waltham, MA, USA) and F-actin was visualized by phalloidin-Alexa488 (1:80; LifeSciences). Final samples were mounted in an anti-fade reagent (ProLong Gold, Thermo Fisher Scientific Inc., Waltham, MA, USA, P36930) for imaging.

Stage 16–17 embryos were fixed and stained as described previously [34]. The following primary antibodies were used: mouse anti-Fasciclin II (1:500; DSHB), anti-BP102 (1:50; DSHB) and anti-DAAM (1:500; [34]). For fluorescent staining, we used Alexa555-anti-mouse and Alexa488-anti-rabbit antibodies. For colorimetric staining, biotin-anti-mouse secondary IgG (Jackson Immunoresearch) was used in combination with a Vectastain ABC kit (Vector Laboratories, Inc., Burlingame, CA, USA) and SigmaFast DAB tablets (Merck KGaA, Darmstadt, Germany).

To analyze the MBs, adult brains were dissected in cold PBS, fixed in 4% paraformaldehyde (diluted in PBS) at RT for 20 min, primary antibody (1:500; mouse anti-FasII, Developmental Studies Hybridoma Bank, Iowa City, IA, USA) was applied overnight (ON) at 4 °C. After the secondary antibody (1:600; Alexa488-anti-mouse) and standard washing steps, samples were mounted in an anti-fade reagent (ProLong Gold, Thermo Fisher Scientific Inc., Waltham, MA, USA, P36930) for imaging.

### 2.7. Schneider 2 Cell Cultures and Transfection

For transfection, 0.25 × 10^6^ S2 cells per well were plated onto 6-well plates and transfected with pAGW, pAGW-CDAAM, pAGW-CDAAM I732A and pAGW-CDAAM FH2 R/K-A constructs using the Effectene transfection reagent (Qiagen). On the following day, 2 h before the fixation, the cells were transferred to a new 6-well plate containing a coverslip pre-coated with concanavalin A. The cells were fixed and stained as the primary neuronal cells, described above. The following primary antibodies were used: mouse anti-α-tubulin (1:1000, DM1A; Merck KGaA, Darmstadt, Germany) and chicken anti-GFP (1:1000, Abcam plc, Cambridge, UK). As secondary antibodies we used chicken Alexa-488 (1:600, Thermo Fisher Scientific Inc., Waltham, MA, USA) and mouse Alexa-546 (1:600, Thermo Scientific).

### 2.8. Microscopy and Image Analysis

Confocal images were captured on a Zeiss LSM800 microscope. Images were then restored by using Huygens Professional Software, 21.10 (Scientific Volume Imaging). Length of the axonal MT bundles was measured by using NeuronJ plugin [37]. MT dynamics were analyzed as described previously [22]. DAB-stained embryos were analyzed on a Zeiss AxioImager M2 microscope.

### 2.9. Statistical Analysis and Figures

Prism 8.0.1 software was used to carry out statistical analysis (GraphPad Software, San Diego, CA, USA). Normality of the data was assessed by D’Agostino–Pearson test. According to the normality, Anova or Kruskal–Wallis test was used for multiple comparisons with either Tukey’s or Dunn’s post hoc test, respectively. Pairwise comparisons were performed by using Student’s *t*-test. In all tests, *p* < 0.05 was considered as statistically significant. Data are represented as mean ± S.D. Figures were prepared by using Illustrator CS6 software (Adobe Inc., San José, CA, USA).

## 3. Results

### 3.1. DAAM and frl Mutant Analysis in Primary Neuronal Cultures

We have previously shown that DAAM has an important role in the development of the embryonic, larval and adult nervous system of Drosophila [28,31,34]. Drosophila DAAM acts as a *bona fide* formin and it is essential for the formation and growth of actin-rich filopodial protrusions [32,34]. DAAM is strongly expressed in the Drosophila embryonic nervous system, as well as in primary neurons, and the lack of DAAM causes axonal growth defects [22,34]. However, DAAM is not the only Drosophila formin that has been linked to CNS development, as we showed that DAAM and FRL regulate axonal growth in the MB of the adult brain in a redundant manner [27]. Since FRL is also expressed in the embryonic CNS and primary neurons (Figure S1A,B), we wanted to clarify its contribution to embryonic axonal development and/or a potential redundancy with DAAM in this context. To this end, we analyzed *DAAM^Ex4^* and *frl^59^* single mutant, and *DAAM^Ex4^;frl^59^* double-mutant embryos. DAAM encodes two major isoforms (PB and PD), of which PB is the predominant one in the embryonic nervous system. DAAM^Ex4^ is a hypomorphic allele which affects the expression of only the PB isoform [28]; therefore, it is an ideal genetic tool to investigate the role of DAAM in axonal growth. Western blot (Wb) analysis showed the DAAM-PB is the only isoform expressed in stage 11 embryos, while DAAM-PD is expressed only at later stages (16–17) (Figure S1C,D). Wb also confirmed that DAAM-PB is completely missing from the *DAAM^Ex4^* embryos, whereas expression of DAAM-PD is not affected (Figure S1C,D). *Frl^59^* is a null mutant allele, generated by CRISPR/Cas9 [29]. Accordingly, Wb analysis showed that the expression of FRL is strongly reduced in the *frl* mutant embryos (Figure S1C,D). *DAAM^Ex4^* and *frl^59^* single mutant homozygous flies are viable, whereas most of the *DAAM^Ex4^;frl^59^* double mutants fail to hatch and die during the larval and pupal stages of development; nevertheless, selection of the double homozygous mutant embryos is straightforward with the help of an appropriate balancer chromosome. 

**Figure 1:**
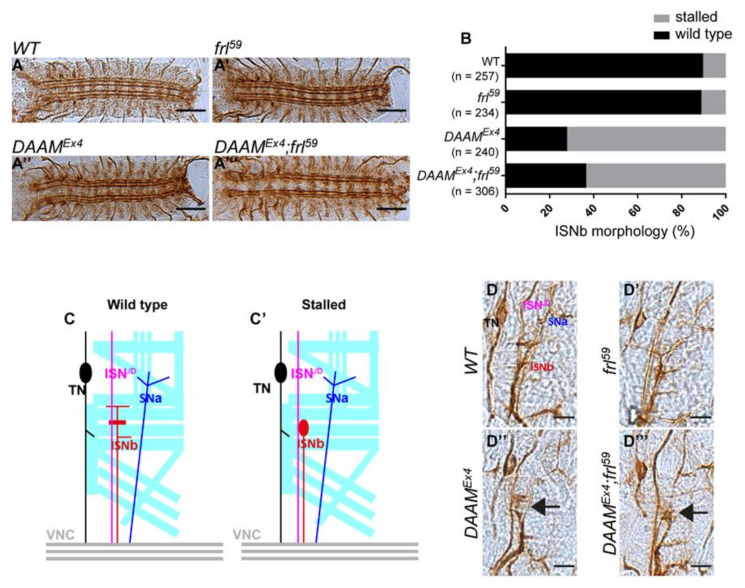
Morphological analysis of the nervous system of *DAAM* and *frl* single- and double-mutant embryos. A) Morphology of FasII-positive motoraxons in the ventral nerve cord of wild-type (**A**), *frl59* (**A′**), *DAAM^Ex4^* (**A″**) and *DAAM^Ex4^/frl^59^* (**A‴**) embryos. Scale bar represents 50 µm. (**B**) Frequency of ISNb phenotypes in wild-type and mutant embryos. (**C**–**C′**) Schematic representation of wild-type and mutant (stalled) motoraxons (TN—transverse nerve, ISNb—intersegmental nerve b, ISNa—intersegmental nerve a, SNa—segmental nerve a). (**D**) Morphology of the FasII-positive motoraxons in the ISNb of wild-type (**D**), *frl^59^* (**D′**), *DAAM^Ex4^* (**D″**) and *DAAM^Ex4^; frl^59^* (**D‴**) embryos. Arrows in (**D″**) and (**D‴**) point to the stalled ISNb. Scale bar represents 10 µm.

**Figure 2:**
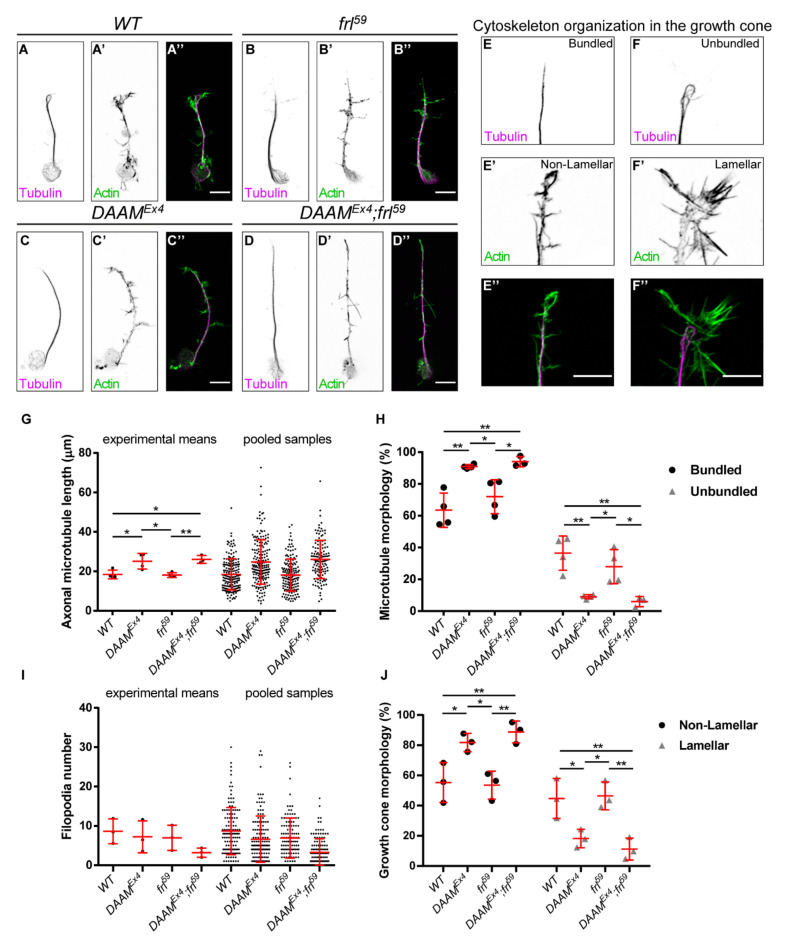
Morphological analysis of primary neurons derived from *DAAM* and *frl* single- and double-mutant embryos. (**A**–**D″**) Representative images of primary neurons derived from wild-type (**A**–**A″**), *DAAM^Ex4^* (**B**–**B″**), *frl^59^* (**C**–**C″**) and *DAAM^Ex4^; frl^59^* (**D**–**D″**) embryos. The actin cytoskeleton was labelled by phalloidin (green); microtubules were visualized by an anti-tubulin (magenta) antibody. Scale bar represents 5 µm. (**E**–**F″**) Examples for cytoskeleton organization of axonal growth cones labelled by phalloidin (green) and anti-tubulin antibody (magenta). Scale bar represents 5 µm. (**G**) Statistical analysis of axonal length of axonal microtubule bundles. Scatter plots show the values of the individual cells and the means of the independent experiments. (**H**) Scatter plots show the frequency of microtubule morphologies in the growth cone. (**I**) Statistical analysis of the axonal filopodia numbers. Scatter plots represent the values of the individual cells and the means of the independent experiments. (**J**) Scatter plots show the frequency of growth cone morphologies labelled by phalloidin. ANOVA was used for statistical analysis. Tukey’s post hoc test was used for multiple comparison. * *p* < 0.05, ** *p* < 0.01.

In order to assess the phenotypic effects of *DAAM* and *frl*, we first examined the morphology of the ventral nerve cord (VNC) in the mutant embryos. Although we found no gross alterations at this level, we observed that development of the intersegmental nerve b (ISNb) was delayed (‘stalled’ phenotype) in *DAAM^Ex4^* and *DAAM^Ex4^; frl^59^* embryos, while the *frl^59^* single mutants appeared normal (Figure 1A–D‴). To extend this analysis to the subcellular level, we generated embryonic primary neuron cultures from stage 11 embryos, as previously described [22]. This was followed by a detailed morphological analysis of neuronal axons, including the measurement of central MT bundle length and filopodia number, and the examination of MT organization and GC morphology (Figure 2A–F″). This analysis revealed that *DAAM^Ex4^* (24.72 ± 11.28 µm, *n* = 187) and *DAAM^Ex4^; frl^59^* (26.04 ± 9.68 µm, *n* = 130) cells have significantly longer central MT bundles as compared to the *frl^59^* single mutants (18.06 ± 8.12 µm, *n* = 160) or wild-type control cells (18.43 ± 7.92 µm, *n* = 173) (Figure 2G). The fact that *DAAM* mutant cells have longer axons is in a good agreement with our previous findings for *DAAM^Ex68^*, a null mutant allele [35]. MT morphology in the GC is significantly altered in both *DAAM^Ex4^* and *DAAM^Ex4^; frl^59^* mutant cells as compared to *frl^59^* single mutant or control cells. Organization of the MTs in the GC was classified either bundled or unbundled (Figure 2E–F″). Unbundled MTs are more spread; they tend to form loops or curves and the occurrence of single MT filaments is more frequent. Conversely, bundled MTs are packed without any individual filaments present. In *DAAM^Ex4^* and *DAAM^Ex4^; frl^59^* neurons, the frequency of bundled MTs is increased, while the frequency of the unbundled MTs is decreased significantly as compared to controls (Figure 2H). Because it is assumed that MT loops indicate a pause of axonal growth [35,38], the increased number of bundled MTs in *DAAM^Ex4^* and *DAAM^Ex4^; frl^59^* neurons is in harmony with the increased length of central MT bundles. Organization of the actin cytoskeleton was studied by counting the number of filopodia and categorizing GC morphology after phalloidin staining. GC morphology was classified as lamellar when GCs were broader with extensive lamellipodia, whereas the non-lamellar GCs appeared pointed at the distal tip of the axons (Figure 2E–F″). Our analysis showed that filopodia number was somewhat decreased in all mutant neurons as compared to control cells, although the difference was not significant in any of the cases (Figure 2I). In contrast, GC morphologies were altered significantly in *DAAM^Ex4^* and *DAAM^Ex4^; frl^59^* mutant cells as compared to *frl^59^* single mutant or control cells (Figure 2J). Non-lamellar GCs tend to have bundled MTs; thus, the frequencies we found are in good correlation with each other, because in *DAAM^Ex4^* and *DAAM^Ex4^; frl^59^* neurons the frequency of bundled MTs and the frequency of non-lamellar GCs are both increased as compared to *frl^59^* single mutant or control cells.

Collectively, these investigations revealed that DAAM is essential for axonal growth in primary neurons, whereas FRL has a negligible or no effect. Analysis of the whole embryonic nervous system pointed towards the same conclusion, as development of the ISNb motoraxons was only impaired in *DAAM^Ex4^* and *DAAM^Ex^; frl^59^* mutants but not in *frl^59^* embryos. These results strongly suggest that, unlike in the adult brain, DAAM and FRL play no redundant roles in the development of the embryonic nervous system. Because of the six Drosophila formins, FHOS, CAPU and Form3 are either not expressed or not required during embryonic CNS development [39], whereas the role of Dia remained unclear; DAAM appears as the most relevant formin with regard to embryonic axonal development. 

### 3.2. Separation of Function Alleles of DAAM

To further understand how DAAM promotes embryonic axon growth, we focused on its potential molecular mechanisms in cytoskeleton regulation. The DAAM protein, similarly to other formins, is able to directly bind actin, as well as microtubules [22,32]. In order to clarify whether actin or MT binding, or both, are required for the proper functioning of DAAM during axonal growth, we created mutations that selectively impair the actin and MT interactions. The FH2 domain of formins forms an antiparallel homodimer and each half of this structure contains two actin-binding sites; one of them (the primary actin-binding site) is marked by a conserved isoleucine (Ile, I) residue, while the other (the secondary actin-binding site) contains a conserved lysine (Lys, K) residue [40,41]. Mutation of these residues strongly reduces the actin-processing activity of several formins [42]. The same result has been reported for Drosophila DAAM, where mutation of the conserved Ile (I732A) residue significantly compromised the actin-processing activity of the FH1-FH2 fragment in vitro [43]. Because of the high-level structural conservation, we expected that a mutation of the secondary actin-binding site (K881A) exhibits a similar effect. More importantly, we formerly demonstrated that the I732A mutant FH2 domain is able to bind MTs in vitro with the same affinity as the wild-type control [22], and for this reason it can be considered as a clean separation of function type of allele.

**Figure 3:**
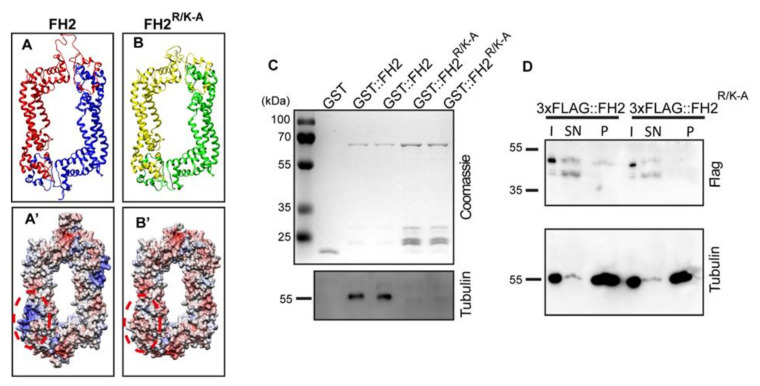
Biochemical characterization of the FH2R/K-A mutant form of DAAM. (**A**–**B′**) Ribbon diagram (**A**,**B**) and surface charge distribution (**A′**,**B′**) of the wild-type and R/K-A mutant FH2 domain of Drosophila DAAM. The region affected by the R/K-A is encircled in red (dashed oval in **A′**,**B′**). (**C**) A GST-pull down assay, carried out by using GST-tagged wild-type and R/K-A mutant FH2 recombinant proteins in combination with purified tubulin. Eluates of the GST-pull down were analyzed by gel electrophoresis and Western blot. Recombinant proteins were visualized by Coomassie blue staining and bound tubulin was detected by an anti-tubulin antibody. Note the presence of tubulin in samples with GST::FH2, and the lack of tubulin when GST::FH2R/K-A was immobilized on the beads. (**D**) Western blot analysis of samples obtained from a microtubule co-sedimentation assay. Tubulin was detected by an anti-tubulin antibody, while the Flag-tagged wild-type and R/K-A mutant form of DAAM FH2 was detected by an anti-Flag antibody. Flag-tagged FH2 protein was detected in the pellet. In contrast, the 3xFLAG::FH2-R/K-A did not co-sediment with microtubules. I—input, SN—supernatant, P—pellet.

**Figure 4:**
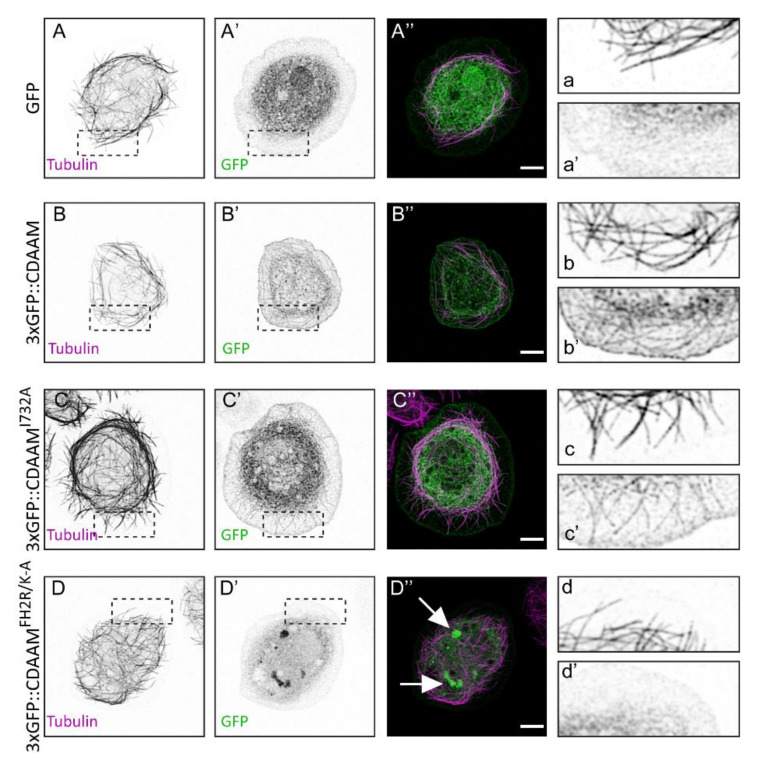
Morphological analysis of S2 cells expressing GFP-tagged wild-type and mutant forms of CDAAM. (**A**–**D″**) Representative images of the cytoskeletal organization of control (**A**–**A″**,**a**,**a′**), CDAAM (**B**–**B″**,**b**,**b′**), CDAAMI732A (**C**–**C″**,**c**,**c′**) and CDAAMFH2R/K-A (**D**–**D″**,**d**,**d′**) expressing S2 cells. GFP or GFP-tagged CDAAM was detected by an anti-GFP antibody (green), and microtubules are visualized by an anti-tubulin antibody (magenta). Filamentous organization of CDAAM was visible in the cortical lamellipodial region of CDAAM and CDAAMI732A expressing cells (see the insets in **b**,**b′**,**c**,**c′,** respectively), which is not present in control cells (**a**,**a′**), and in cells expressing CDAAMFH2R/K-A (**d**,**d′**) where most of the GFP signal accumulates into cytoplasmic foci (arrows in **D″**). Scale bar represents 5 µm.

As to MT binding, we have shown that Drosophila DAAM directly interacts with MTs via its FH2 domain and the C-terminal “tail” region [22]. Given that the CT region is much shorter, and the CT mutations we previously tested affected the interaction with both cytoskeletal components [43], we focused on the FH2 domain and aimed to create an FH2 mutant version with reduced MT-binding capacity and unimpaired actin-processing activity. Structural and bioinformatics analysis of the FH2 domain identified a potential binding region for the negatively charged MT surface. The positive charge of this patch results from the presence of five basic amino acids in a seven-amino-acid-long motif containing amino acids 840–846 (Arg-Ser-Arg-Arg-Leu-Arg-Lys). A mutant form of the DAAM FH2 domain was created, in which these basic amino acids were replaced with Alanine (FH2^R/K-A^). Subsequent surface charge calculations showed that the previously identified positive patch disappeared in the mutant protein (Figure 3A–B′). GST pull-down and MT co-sedimentation assays revealed that in vitro the FH2^R/K-A^ version has a significantly reduced tubulin- and MT-binding capacity as compared to wild-type FH2 (Figure 3C,D). This effect has been further confirmed in Drosophila S2 cells, where we found that GFP-tagged versions of the wild-type and I732A mutant CDAAM (a truncated form consisting of the FH1-FH2-DAD-CT regions) exhibit a strong colocalization with MTs (Figure 4A–C″), that is largely reduced in the case of CDAAM^R/K-A^ (Figure 4D–D″). To test for actin assembly, we attempted to purify the CDAAM^R/K-A^ mutant protein; however, this protein has a tendency to form aggregates in vitro resulting in low-quality preparations. We observed a similar behavior in S2 cells where CDAAM and CDAAM^I732A^ exhibited a largely uniform subcellular distribution around the nuclei and in the cortical, lamellipodial region (Figure 4B–C″), whereas the CDAAM^FH2R/K-A^ mutant protein often accumulated into huge cytoplasmic and nuclear puncta (Figure 4D–D″). Because of this difficulty, it is not possible to reliably conclude on the actin assembly activity of CDAAM^FH2R/K-A^. Nevertheless, it is noteworthy that when present in DAAM-PB, the R/K-A mutation does not impair the in vivo activity of DAAM in primary neurons (see below), indicating that at least in context of the full-length protein, this mutation is very unlikely to interfere with actin polymerization. 

### 3.3. The Actin-Processing Activity of DAAM Is Essential for Axon Growth

**Figure 5:**
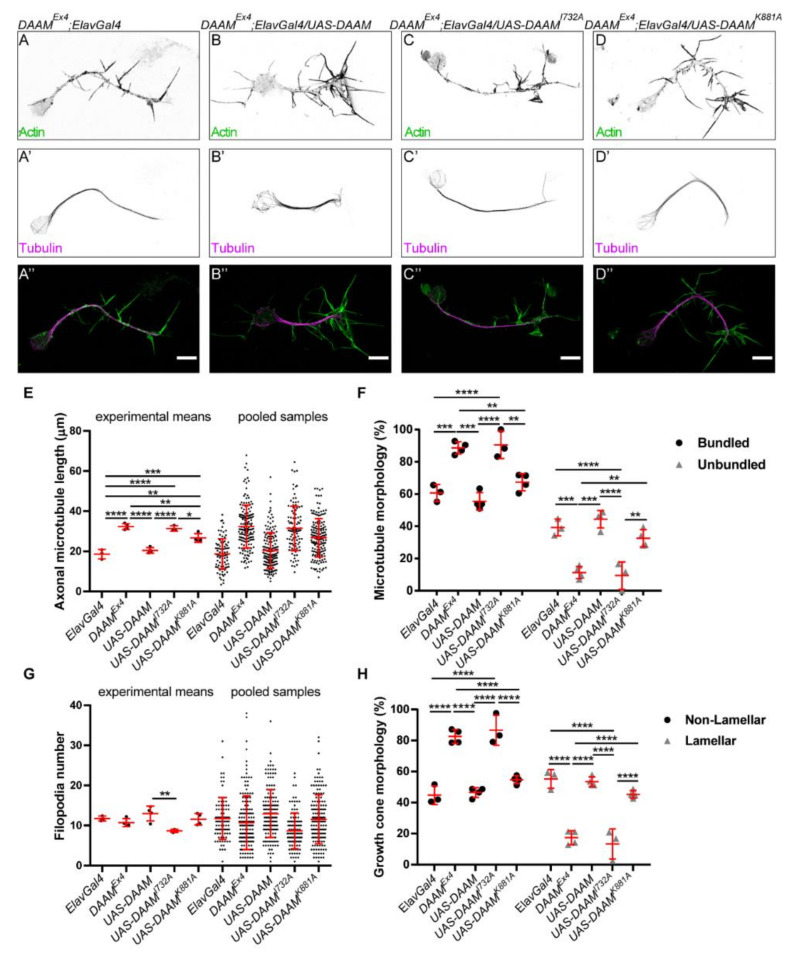
The actin-processing activity of DAAM plays a role in axon development in primary neurons. (**A**–**D″**) Representative images of the axonal and growth cone morphology of primary neurons derived from *DAAM^Ex4^;Elav-Gal4* control (**A**–**A″**) and transgene expressing (**B**–**B″**-*DAAM*; **C**–**C″**-*DAAM^I732A^*; **D**–**D″**-*DAAM^K881A^*) *Drosophila* embryos. The actin cytoskeleton was labelled by phalloidin (green), and microtubules were detected by an anti-tubulin antibody (magenta). Scale bar represents 5 µm. (**E**) Statistical analysis of the length of the axonal microtubule bundles. Scatter plots show the values of the individual cells and the means of the independent experiments. (**F**) Scatter plots show the frequency of microtubule morphologies in the growth cone. (**G**) Statistical analysis of the axonal filopodia numbers. Scatter plots represent the values of the individual cells and the means of the independent experiments. (**H**) Scatter plots show the frequency of growth cone morphologies labelled by phalloidin. ANOVA was used for statistical analysis. Tukey’s post hoc test was used for multiple comparison. * *p* < 0.05, ** *p* < 0.01, *** *p* < 0.001, **** *p* < 0.0001.

After basic biochemical characterization of the FH2 domains with the separation of function mutations, we created transgenic flies that carry the corresponding mutations in a UAS-DAAM-PB construct. First, we studied the rescue ability of the actin-processing mutants (I732A and K881A) by expressing them in *DAAM^Ex4^* mutant embryos by using the pan-neuronal ElavGal4 driver. Interpretation of the rescue experiments can be misleading without having data on the expression levels and patterns of the transgenes. To exclude this problem, Wb and immunostaining experiments were performed, and they showed that all of the transgenes were expressed at equal levels and the proteins could be detected specifically in the embryonic nervous system (Figure S2A–F). Moreover, Wb also showed that the ElavGal4 driver was turned on at stage 11, when the embryos were collected to prepare primary cultures and expressions of the transgenes were getting stronger over time (Figure S2A). Primary neurons were extracted from embryos expressing either the wild-type or the actin-processing mutant forms of *DAAM* (Figure 5A–D″). Morphological analysis revealed that primary neurons expressing the wild-type UAS-DAAM-PB isoform had significantly shorter (20.43 ± 8.85 µm, *n* = 162) central MT bundles in their axons as compared to control mutant cells (32.36 ± 10.68 µm, *n* = 161) carrying only the ElavGal4 construct, but no transgene (Figure 5E). In contrast, axonal length of neurons expressing DAAM-PB^I732A^ did not show any difference (31.46 ± 11.06 µm, *n* = 114), whereas cells expressing DAAM-PB^K881A^ displayed a medium, but significant, decrease (26.74 ± 9.56 µm, *n* = 161) as compared to mutant cells (Figure 5E). Analysis of MT and actin cytoskeleton organization in the GCs indicated that expression of the wild-type DAAM-PB fully rescued the MT and GC morphology defects exhibited by the *DAAM^Ex4^* mutant cells, whereas expression of DAAM-PB^I732A^ failed to provide a rescue, while DAAM-PB^K881A^ was able to partially restore the wild-type phenotype in mutant cells (Figure 5F,H). Filopodia numbers did not considerably change in any of the genotypes, although there was a significant difference between wild-type and I732A-mutant-DAAM-expressing neurons (Figure 5G).

Together, these rescue experiments revealed that the I732A mutant form of DAAM, impaired in its actin assembly activity, is not able to compensate for the lack of the wild-type protein at all. Therefore, it appears likely that presence of the I732A mutation makes the DAAM protein completely or largely dysfunctional, and that the actin-processing activity of DAAM is indispensable for proper axonal differentiation. Interestingly, disruption of the secondary actin-binding pocket of DAAM by the K881A mutation was still able to partially restore the wild-type phenotype, suggesting that in the case of DAAM, the secondary actin-binding surface of the FH2 domain is not absolutely required for function.

### 3.4. Direct MT-Binding of DAAM Is Not Essentially Required for Axon Growth

**Figure 6:**
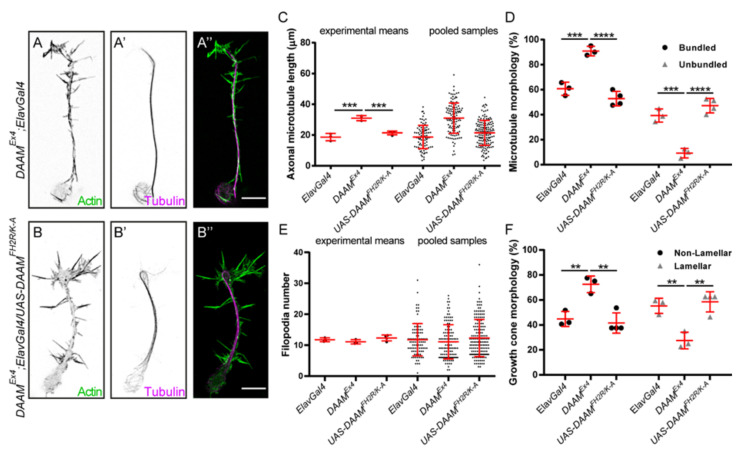
Morphological analysis of primary neurons derived from *DAAM^Ex4^* mutant *Drosophila* embryos expressing *DAAM^FH2R/K-A^*. (**A**–**B″**) Representative images of the axonal and growth cone morphology of primary neurons derived from *DAAM^Ex4^;Elav-Gal4* control (**A**–**A″**) and *DAAM^FH2R/K-A^* (**B**–**B″**) expressing *Drosophila* embryos. The actin cytoskeleton was labelled by phalloidin (green), and microtubules were detected by an anti-tubulin antibody (magenta). Scale bar represents 5 µm. (**C**) Statistical analysis of the length of axonal microtubule bundles. Scatter plots show the values of the individual cells and the means of the independent experiments. (**D**) Scatter plots show the frequency of microtubule morphologies in the growth cone. (**E**) Statistical analysis of the axonal filopodia numbers. Scatter plots represent the values of the individual cells and the means of the independent experiments. (**F**) Scatter plots show the frequency of growth cone morphologies labeled by phalloidin. ANOVA was used for statistical analysis. Tukey’s post hoc test was used for multiple comparison. ** *p* < 0.01, *** *p* < 0.001, **** *p* < 0.0001.

Several studies established that formins can directly interact with MTs and may have a regulatory role in MT dynamics [15,17,18,19]. We have also shown that Drosophila DAAM directly interacts with MTs in in vitro assays; DAAM is able to stabilize the MTs in vitro and to protect them from MT de-stabilizing drugs in primary neurons, and it can crosslink and/or co-align the MTs with those of F-actin [22]. To probe the functional importance of direct MT binding, we made use of the UAS-DAAM-PB^FH2R/K-A^ transgene, expressing a mutant form with reduced MT-binding capacity, in rescue experiments. Similar to the previous set of rescue experiments, the UAS-DAAM-PB^FH2R/K-A^ transgene was expressed in primary neurons by using the ElavGal4 driver line (Figure 6A–B″). Immunostaining and Wb experiments showed that this transgene ensured comparable expression levels as to the I732A and K881A mutant lines (Figure S3A–C′). Morphological analysis of *DAAM^Ex4^* mutant primary neurons expressing DAAM-PB^FH2R/K-A^ revealed that this form of DAAM nearly perfectly restored the wild-type phenotype. Length of the central axonal MT bundles was significantly decreased in DAAM-PB^FH2R/K-A^ expressing neurons (21.39 ± 8.11 µm, *n* = 161) as compared to control mutant cells (30.95 ± 9.88 µm, *n* = 120) and became similar to that of wild-type cells (Figure 6C). Accordingly, the frequency of the GCs with bundled MTs and non-lamellar morphology was reduced significantly (Figure 6D,F), and again became much like the numbers measured in wild-type. Just as in the case of the other transgenes used during these studies, filopodia number was not significantly affected by the presence of DAAM-PB^FH2R/K-A^ (Figure 6E). The fact that the DAAM-PB^FH2R/K-A^ transgene exhibited a highly similar rescue ability as the wild-type transgene indicates that the MT-binding capacity of DAAM has negligible contribution to its function in cultured embryonic primary neurons. 

**Figure 7:**
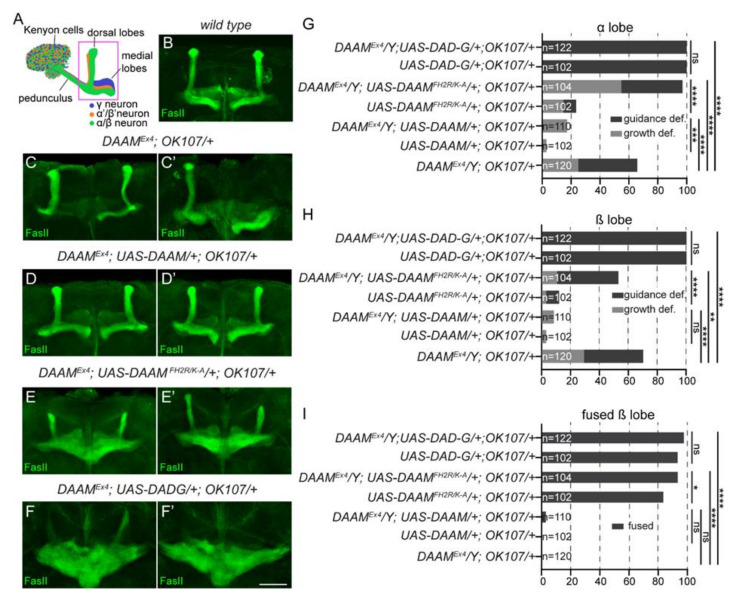
The *FH2R/K-A* mutant form of *DAAM* fails to rescue the MB axonal defects observed in *DAAM^Ex4^*. (**A**) Schematic representation of the organization of the mushroom bodies. (**B**–**F′**) Confocal images of MBs stained for FasII (green) to label the α and β-lobes; with the exception of wild-type; (**B**) two representative examples are shown for each genotype. The *DAAM^Ex4^* mutant (**C**,**C′**) MBs exhibit various defects in axonal development resulting in missing, shorter, thinner or thicker lobes, which can be perfectly rescued by *UAS-DAAM* expression (**D**,**D′**). As compared to this, the expression of *UAS-DAAM^FH2R/K-A^*, in addition to various effects on the dorsal lobes, results in overprojection of the β axons (**E**,**E′**) which is very similar to the effect of *DAD-G* expression (**F**,**F′**). (**G**) Quantification of the axonal growth and guidance and defects detected in the MB α-lobes of adults of the genotypes indicated. (**H**) Quantification of the axonal growth and guidance and defects detected in the MB β-lobes of adults of the genotypes indicated. (**I**) Quantification of the β-lobe fusion phenotype in the MBs of adults of the genotypes indicated. Chi-square or Fisher’s exact tests were used for statistical analysis. * *p* < 0.05, ** *p* < 0.01, *** *p* < 0.001, **** *p* < 0.0001, ns—not significant. Scale bar represents 50 µm.

Previously we reported that, in addition to the role in embryonic axonal growth, DAAM plays a pivotal role in axonal growth and guidance of the adult mushroom body (MB) neurons [28]. In order to address the in vivo effect of DAAM-PB^FH2R/K-A^, rescue experiments were performed in the MB of the *Drosophila* adult brain. The MB is a central brain region formed by three major classes of neurons, the γ, α′/β′ and α/β neurons, each characterized with a well-defined axonal projection pattern (Figure 7A). Because axons of the α′/β′ and α/β neurons are bifurcated, the MB consists of five bundled axons that organize into the vertical α, α′ and the medial β, β′ and γ lobes. As shown before, the *DAAM^Ex4^* mutant flies exhibit strong axonal growth and guidance defects in the MB that we quantified in the α/β neurons (Figure 7B–C′,G,H). Whereas OK107-Gal4 driven MB-specific expression of the wild-type DAAM-PB rescued the mutant phenotype (Figure 7D,D′,G,H), the DAAM-PB^FH2R/K-A^ transgene failed to do so (Figure 7E,E′,G,H). Notably, in MBs of *DAAM^Ex4^*; *UAS- DAAM-PB^FH2R/K-A^*/*+*; *OK107-Gal4*/*+* mutant flies, the α-lobes were either shorter or thinner than in wild-type, while the β-lobes were either normal or thicker (Figure 7E,E′), indicating that growth of the α-axons is repressed whereas growth of the β-axons is promoted by the presence of the DAAM-PB^FH2R/K-A^ transgene. In line with this, careful examination of the lobes revealed that expression of DAAM-PB^FH2R/K-A^ resulted in a β-lobe fusion phenotype when the β-axons erroneously cross the CNS midline (Figure 7E,E′,I). This effect highly resembles the phenotype caused by overexpression of a constitutively active form of DAAM (DAD-G) lacking the C-terminal autoinhibitory domain (Figure 7F,F′,I). As the β-lobe fusion phenotype is interpreted as a gain-of-function (GOF) effect due to overextension of the β-axons, these findings are in harmony with the primary neuronal data suggesting that the DAAM-PB^FH2R/K-A^ mutant form is active in vivo, and very unlikely to be impaired in its actin assembly activity. Together, the plethora of defects affecting MB axonal development imply that the *FH2R/K-A* mutation alters the regulation of DAAM activity in the MB. Thus, MT side-binding may not play a significant role in execution of the MT-related formin functions in cultured neurons, yet it appears to be important for proper MB axon formation in vivo. 

### 3.5. Overexpression of a Constitutively Active Form of DAAM

**Figure 8:**
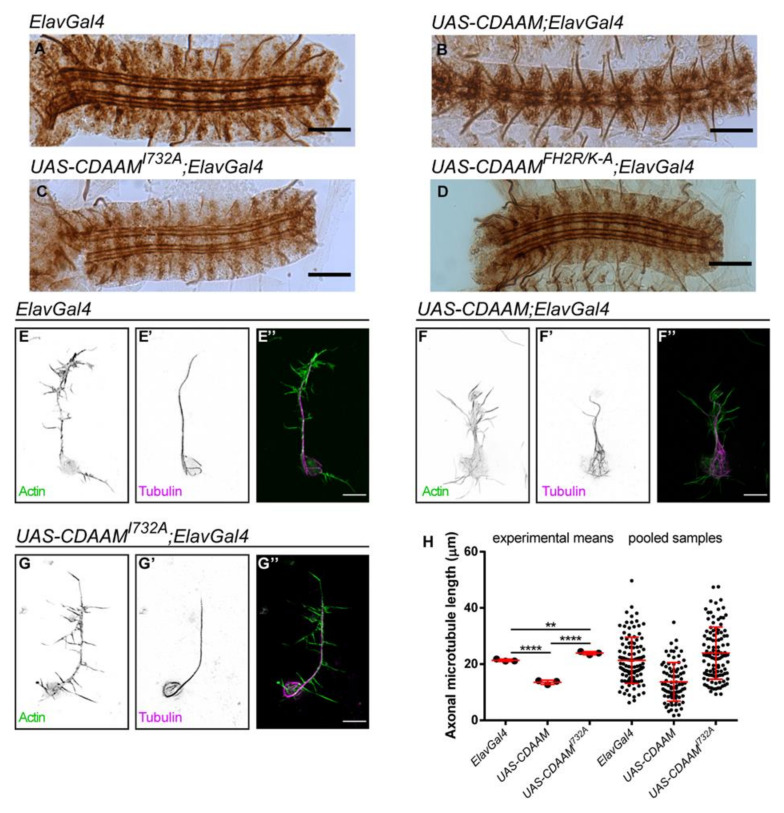
The effect of CDAAM overexpression on neuronal morphology. (**A**–**D**) Morphology of the FasII-positive motoraxons in the ventral nerve cord of control (*Elav-Gal4*) (**A**), *CDAAM* (**B**), *CDAAM^I732A^* (**C**) and *CDAAM^FH2R/K-A^* (**D**) expressing *Drosophila* embryos. Scale bar represents 50 µm. (**E**–**G″**) Representative images of axonal and growth cone morphology of primary neurons derived from control (**E**–**E″**), *CDAAM* (**F**–**F″**) or *CDAAM^I732A^* (**G**–**G″**) expressing *Drosophila* embryos. The actin cytoskeleton was labelled by phalloidin (green), and microtubules were detected by an anti-tubulin antibody (magenta). Scale bar represents 5 µm. (**H**) Statistical analysis of the length of axonal microtubule bundles. Scatter plots show the values of the individual cells and the means of the independent experiments. ANOVA was used for statistical analysis. Tukey’s post hoc test was used for multiple comparison. ** *p* < 0.01, **** *p* < 0.0001.

To further test the effect of the separation of function alleles, we employed a GOF system based on overexpression of the constitutively active CDAAM form. When CDAAM is expressed in embryonic neurons, it strongly interferes with axonal growth evident in the development of the embryonic CNS as well as in primary neurons [22,34]. To address whether the GOF effect of CDAAM depends on actin-binding or MT side-binding activities, we investigated the effect of the I732A and *FH2R/K-A* mutation on the activity of CDAAM. The wild-type and mutant forms of UAS-CDAAM were expressed in the embryonic CNS by using ElavGal4. Expression of the transgenes was checked by Wb experiments, revealing comparable levels in each case (Figure S2B). Analysis of whole embryos showed that CDAAM overexpression strongly disrupts the organization of Fasciclin II (FasII)-positive motoraxons in the VNC, while embryos expressing the I732A mutant form of CDAAM did not display any aberration in the nervous system (Figure 8A–C). Morphological analysis of primary neurons showed that cells overexpressing CDAAM had a significantly shorter (13.69 ± 6.87 µm) central MT bundle in their axons as compared to control cells (21.33 ± 8.22 µm) (Figure 8E,F″,H). In contrast, overexpression of CDAAM^I732A^ had no striking effect on axonal growth (23.91 ± 9.27 µm) (Figure 8E–E″,G–G″,H). Live imaging experiments were also performed expressing an EB1::GFP construct in primary neurons (Figure S4A) to investigate MT dynamics in living cells [44]. We found that the velocity of MT growth was reduced in neurons overexpressing wild-type CDAAM, whereas the overexpression of *CDAAM^I732A^* did not alter MT dynamics as compared to control cells (Figure S4B). 

As compared to CDAAM^I732A^, the CDAAM^FH2R/K-A^ mutant form exhibited nearly identical phenotypic effects, and overall, the *FH2R/K-A* mutation appeared to be a potent suppressor of the CDAAM GOF effects (Figure 8D). This would argue that, beyond actin binding, MT side-binding is an equally important aspect of the mechanism whereby CDAAM interferes with axonal growth. However, it is notable that CDAAM has only a moderate effect on MT growth speed (Figure S4), suggesting that the effect of CDAAM on MT dynamics might be limited. In addition, our in vitro data with the purified CDAAM^FH2R/K-A^ mutant hinted at the possibility of inactivation due to protein aggregation that also happens in vivo in S2 cells. Collectively, these results clearly revealed that the presence of the I732A mutation completely abolishes the GOF effect of CDAAM, indicating an actin dependence. Contrasting to this, the contribution of MT side-binding remained more controversial, and further clarifications will require investigation of the structural consequences of the R/K-A mutation. 

## 4. Discussion

Being important cytoskeleton regulators, several formins proved to be essential during the development of the nervous system [45]. Previously, we have shown that DAAM plays a role in the development of the Drosophila nervous system at different developmental stages from embryo to adulthood [22,28,31,34]. In addition, DAAM cooperates with FRL during axon formation in the mushroom bodies of the adult brain [27]. In this paper, we investigated the possible redundant role of DAAM and FRL during the development of the embryonic nervous system. The analysis of single and double mutants showed that FRL has a negligible effect on axonal growth in primary neurons and development of the Fas II-positive motoraxons in embryos, and therefore the CNS function of FRL seems restricted to the adult stage. In contrast, the effect of the lack of DAAM was similar to what had been observed in previous studies [34,35]. As RNA in situ hybridization data and single-mutant analysis argue against an embryonic CNS function in the cases of Fhos, Capu and Form3 [39], our results clearly suggest DAAM being the main formin in the Drosophila embryonic CNS. Nevertheless, whether DAAM is redundant with another formin than FRL remained an open question as double-mutant analysis is not reported for *fhos*, *capu* and *form3*, nor for *dia* where a further level of complexity arises due to its role in cytokinesis. 

The process of axonal growth is driven by coordinated changes of the actin- and MT-cytoskeleton. Several formin proteins have been shown to be important in actin–MT crosstalk, and besides their general role in actin regulation, some formins also have an actin-independent MT-related activity. Recent studies revealed that formins induce the formation of stable MTs and/or play a role in crosslinking of actin and MT filaments in different neuronal model systems [23,42,43]. Interestingly, these studies involved several formins (mDia1, Fmn2, and Drosophila Form3 ortholog of mammalian INF2) belonging to different subfamilies, which strongly suggests that an MT-related activity might be a general feature of formins expressed in the nervous system. Consistent with these findings, we have also shown that in the absence of DAAM, MT dynamics, especially the velocity of MT growth, was significantly increased in mutant neuronal cells [22]. We also showed that DAAM can directly interact with MTs, and it protects MTs from cold-induced depolymerization in vitro and stabilizes MTs in nocodazole-treated primary neurons [22]. Despite these advances, molecular mechanisms of the formin-mediated actin–MT crosstalk and that of MT cytoskeleton regulation remained largely unclear. To address this issue, we reasoned that the employment of mutations that selectively affect the actin- or the MT-binding ability of DAAM would be beneficial to test. The I732A mutation was used as an actin-processing mutant, and we found that it is not able to rescue the mutant phenotype of primary neurons. Since the I732A mutation does not disturb the MT-binding capacity of DAAM [22], these experiments clearly show that the actin-processing activity of DAAM is indispensable for axon growth. We also wanted to perform the rescue experiment by using a mutant with the opposite features, where MT-binding is reduced but the actin-processing function is not disturbed. However, due to the fact that the actin- and MT-binding surfaces overlap in DAAM, it is a challenging task to create such a mutant form of the protein. Based on protein interaction studies, the FH2 domain has been identified as the chief MT-binding surface of formins [17,18,22], yet the molecular details were not resolved. It is very likely that neither the conserved Ile in the middle of the primary actin-binding site nor the dimeric structure of the FH2 domain is necessary for MT-binding [17,22]. Besides the FH2 domain, several formins carry an additional MT-binding surface in their CT region, which is highly basic; therefore, it is an ideal candidate for electrostatic interactions with the negatively charged MT surfaces [18,19,23]. The specific MT-binding domain has been mapped for only two formins, inverted formin 1 (INF1) and formin 1 [15,16]. Our previous studies showed that Drosophila DAAM binds to MTs via its FH2 and CT regions [22]. Due to the fact that, besides the FH2 domain, the CT region also has a role in actin binding and F-actin assembly [43], and separation of the actin versus MT-linked functions in the CT region appears extremely difficult. In this study, we identified a short, positively charged motif in the FH2 domain of DAAM, which is important in both tubulin- and MT-binding in vitro as suggested by our GST pull-down and MT co-sedimentation assays. Due to the presence of the CT region, mutation of this short motif in the FH2 domain is unlikely to completely abolish the MT-binding capacity of the full-length DAAM protein, but it is very likely that at least one of the MT-binding surfaces would be compromised. When this mutant form (R/K-A) was probed for rescue in embryos, expression of this protein fully restored the wild-type axonal phenotype of the neurons. However, when the rescue ability was tested in the adult brain, the UAS-DAAM^FH2R/K-A^ construct failed to provide rescue in the MB axons; instead, it induced a β-axon overextension phenotype, typically observed upon DAAM overactivation. This specific defect in axonal guidance might indicate that a DAAM–MT association is critical to steer axons into the right direction, at least in the MB neurons. Together, these results demonstrate that the R/K-A mutant form is fully active, and the MT side-binding capacity of DAAM through this motif is not essential for axonal growth in cultured primary neurons, and as inferred from this, in the embryonic CNS. Because of the presence of the CT region and the possibility of indirect MT binding (see below), we emphasize that these data do not rule out the importance of formin MT binding in general, it only addresses the functional importance of a newly identified motif. Interestingly, it appears that in MB neurons, the R/K-A mutant behaves similar to a constitutively active form, suggesting that the mutant residues are involved in the regulation of DAAM activity. Whether this is an MT-dependent phenomenon, or the mutations induce a structural alteration that partly impairs the formin-autoinhibitory mechanism or binding to an MB-specific regulatory factor, awaits future elucidations.

In contrast to the rescue experiments carried out with the full-length DAAM protein and where the I732A and the R/K-A mutants exhibited distinct effects, in the context of the truncated, constitutively active CDAAM form, both mutations were able to suppress the GOF effect. The strong influence of I732A is consistent with the rescue data, and importantly, it reveals that the GOF effect of CDAAM critically depends on its actin-processing activity. Curiously, although the R/K-A mutations do not abolish the activity of the full-length DAAM protein, neuronal expression of the CDAAM^FH2R/K-A^ mutant form does not result in a GOF effect. One interpretation of this observation would be that, besides actin interaction, MT side-binding is also essential for the GOF effect of CDAAM. Whereas it would be an interesting scenario if the R/K-A mutations would indeed exert a differential effect in context of the full-length protein as compared to the truncated version, caution might be required with this interpretation as in vitro behavior of the CDAAM^FH2R/K-A^ mutant protein indicated a tendency for aggregation, also observed upon CDAAM^FH2R/K-A^ expression in S2 cells. 

Regarding the potential molecular mechanisms of the formin-mediated actin/MT cytoskeleton coordination during axonal growth, so far, we have mainly considered the ones that involve direct protein interactions. Indeed, it has been shown for several formins that they can simultaneously interact with actin and MT filaments, and they can crosslink the two filamentous systems [18,19,22,23]. Importantly, however, in addition to the direct interactions, formins were shown to be able to bind to a relatively large number of cytoskeleton regulatory proteins, and these interactions have a large impact on the cytoskeleton coordinator function of formins. For example, some formins can interact with MT + TIPs, which in turn induces cytoskeleton rearrangements and influences the activity of formins [22,26,46]. Formins can also induce the stabilization of MTs by inducing the acetylation of the lysine-40 residue of α-tubulin and they have the ability to form stable Glu MTs [20,21]. Moreover, in human retinal epithelial cells, the MT-stabilizing activity of INF2 relies on hierarchically organized protein complexes or pathways [45], and it was shown that the I732A equivalent mutant reduces the tubulin acetylation activity of INF2. Collectively, these data highlight multiple indirect mechanisms whereby formin proteins could play a role in neuronal cytoskeleton coordination. Thus, although we found that the direct MT-binding ability of DAAM is less important in differentiation of the Drosophila primary neurons, to fully explore the significance of the formin-mediated MT and cytoskeleton crosslinking functions in axons, further studies will be required by systematic analysis of the +TIP interactions and the potential signaling pathways. Likewise, it would be of interest to test whether the actin polymerization of incompetent formins affects tubulin acetylation in axonal growth cones. 

## Data Availability

The datasets generated and/or analyzed during the current study are available from the corresponding author upon request.

## Supplementary Materials

The following supporting information can be downloaded at: https://www.mdpi.com/article/10.3390/cells11091487/s1, Figure S1: Analysis of the expression of DAAM and FRL in wild type and formin mutant embryos and primary neurons; Figure S2: Analysis of the expression level of full length DAAM and CDAAM transgenes upon Elav-Gal4 driven expression; Figure S3: Analysis of the expression of DAAMFH2^R/K-A^ in *DAAM^Ex4^* mutant embryos; Figure S4: Live imaging analysis of microtubule growth in control and CDAAM expressing primary neurons; Table S1: List of primers used for generation of the FH2^R/K-A^ mutant.
