# Supplementary material for: Molecular Dissection of DAAM Function during Axon Growth in Drosophila Embryonic Neurons
Source: Cells. 2022 Apr 28;11(9):1487. doi: 10.3390/cells11091487 (PMC9102401; doi:10.3390/cells11091487)
Supplement: Supplementary file 1 [file cells-11-01487-s001.zip › cells-1691757-supplementary.pdf]

Supplementary Materials

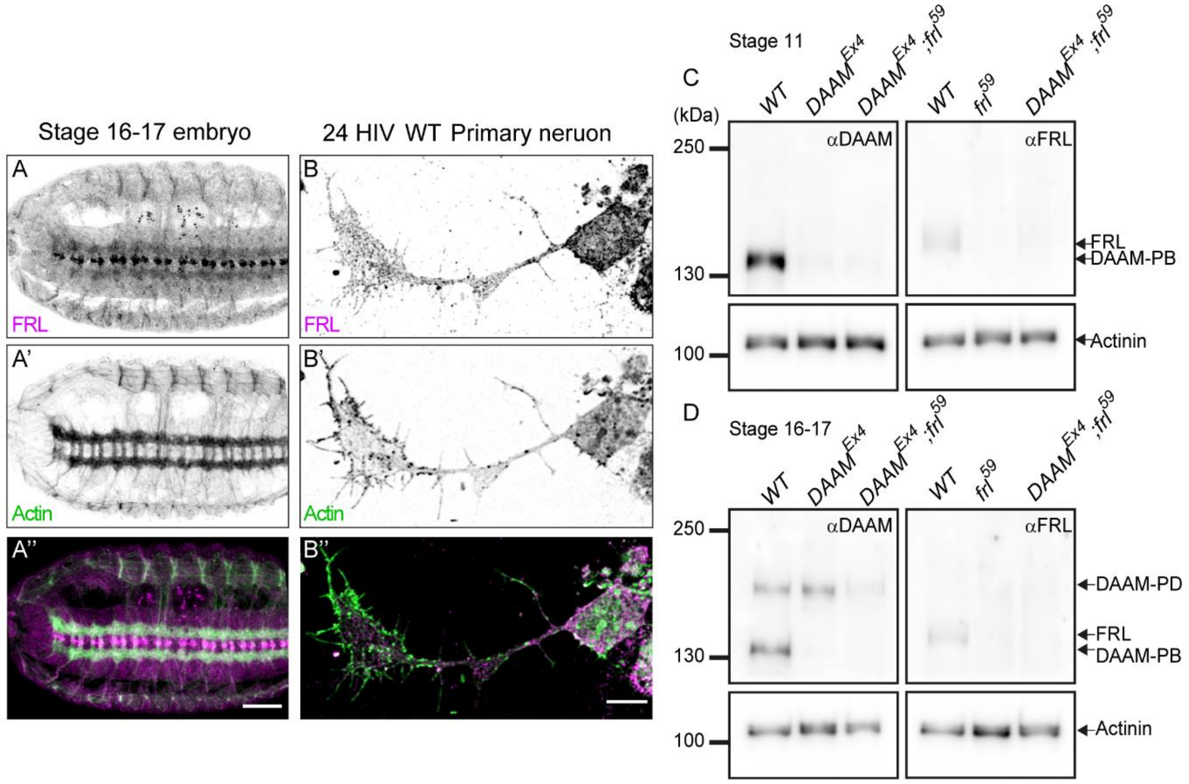

Supplementary Figure S1. Analysis of the expression of DAAM and FRL in wild type and formin mutant embryos and primary neurons.
(A-B”) Immunostaining of stage 16-17 wild type embryos (A-A``) and primary neurons (24 HIV) (B-B``) shows that FRL is expressed in the embryonic central nervous system and in the axons of primary neurons, respectively. FRL was detected by an anti-FRL antibody, actin was labelled by phalloidin. Scale bars: 50 µm (A-A”) and 5 µm (B-B”). (C,D) Western blot analysis shows that the DAAM and FRL proteins are present in the wild type embryos, but they cannot be detected in the corresponding mutant embryos in stage 11 (C) and stage 16-17 (D). DAAM was detected by an anti-DAAM, FRL was detected by an anti-FRL, Actinin (detected by an anti-actinin antibody) was used as a loading control.

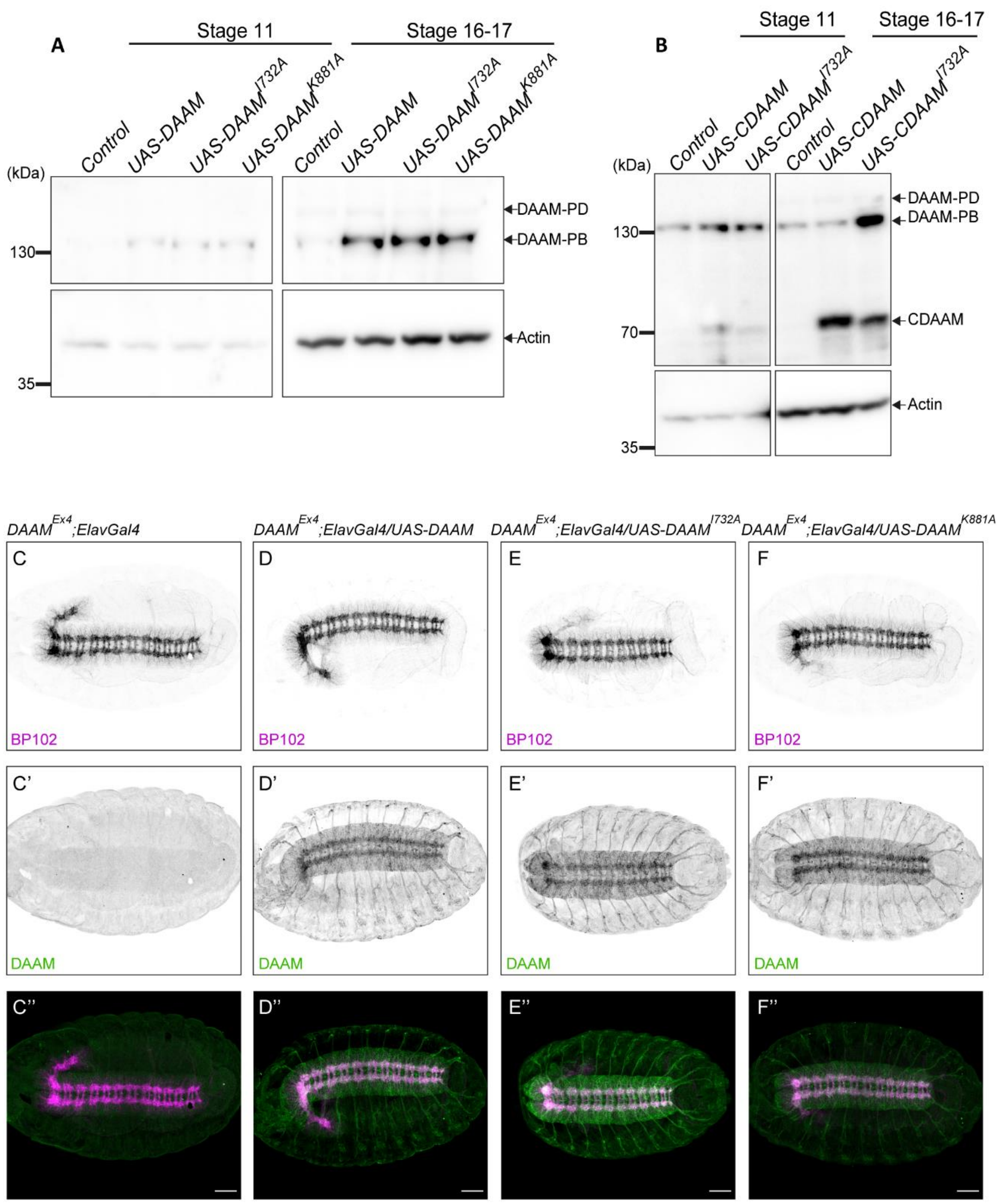

Supplementary Figure S2. Analysis of the expression level of full length DAAM and CDAAM transgenes upon Elav-Gal4 driven expression.

(A) Western blot analysis shows that DAAM, DAAM^{I732A} and DAAM^{K881A} are expressed at a comparable level in stage 11 and stage 16-17 embryos when expressed with *Elav-Gal4* in *DAAM^{Ex4}* mutants (used as control). DAAM was detected by an anti-DAAM; actin (detected by an anti-actin antibody) was used as a loading control. (B) Western blot shows that the UAS-CDAAM and UAS-CDAAM^{I732A} transgenes are expressed at a comparable levels in stage 11 and stage 16-17 embryos when expressed with *Elav-Gal4* (used as control). CDAAM was detected by an anti-DAAM; actin (detected by an anti-actin antibody) was used as a loading control. (C-F'') Immunostaining of stage 16-17 embryos shows that DAAM is not present in

DAAM^{Ex4} animals (C-C``), whereas the *Elav-Gal4* driven expression of DAAM (D-D``), DAAM^{I732A} (E-E``) and DAAM$^{K881A}$ (F-F``) in a *DAAM* mutant background results in expression that can be detected in both the central and peripheral nervous system. DAAM is detected by an anti-DAAM antibody (green). BP102 staining was used to mark the axon bundles in the ventral nerve cord (magenta). Scale bar represents 50 µm.

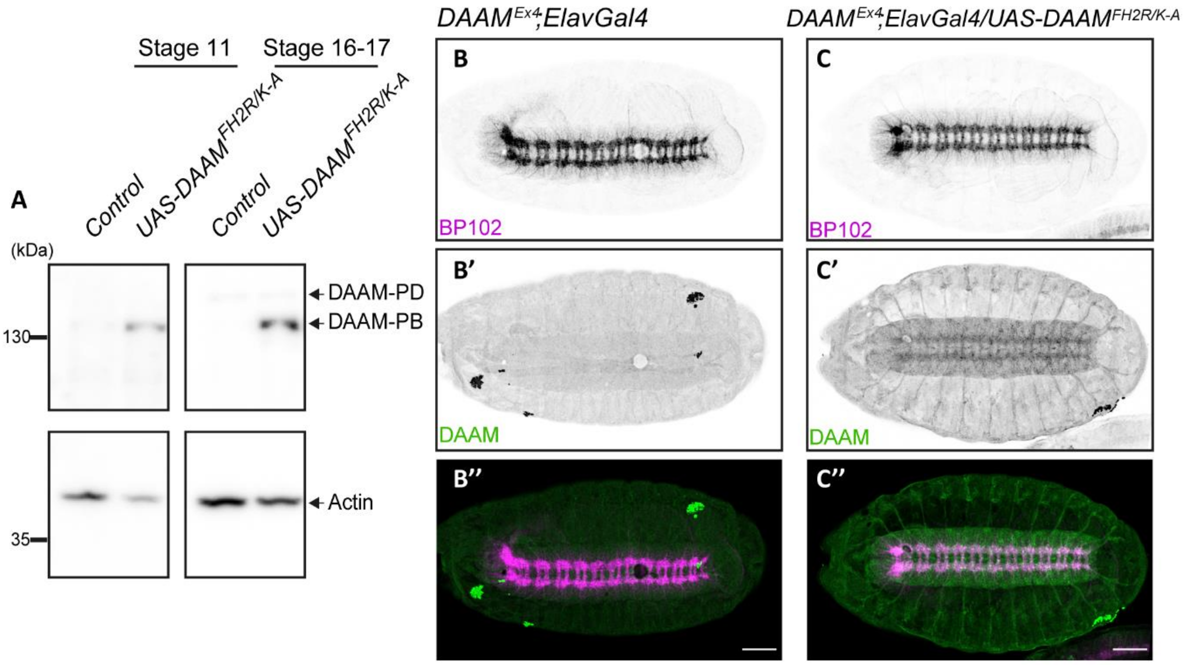

Supplementary Figure S3. Analysis of the expression of DAAM$^{FH2R/K-A}$ in *DAAM*^{Ex4} mutant embryos.

(A) Western blot analysis shows that *Elav-Gal4* driven expression of the DAAM$^{FH2R/K-A}$ mutant form in a *DAAM*^{Ex4} background can be clearly detected in stage 11 and stage 16-17 embryos. *DAAM*^{Ex4}; *Elav-Gal4* embryos were used as control. DAAM was detected by an anti-DAAM, actin (detected by an anti-actin antibody) was used as a loading control. (B-C”) Immunostaining of stage 16-17 embryos show that DAAM is not present in *DAAM*^{Ex4} animals (B-B``), whereas expression of the DAAM$^{FH2R/K-A}$ mutant protein (C-C``) is evident in the central and peripheral nervous system. DAAM is detected by anti-DAAM antibody (green). BP102 staining was used to mark the axon bundles in the ventral nerve cord (magenta). Scale bar represents 50 µm.

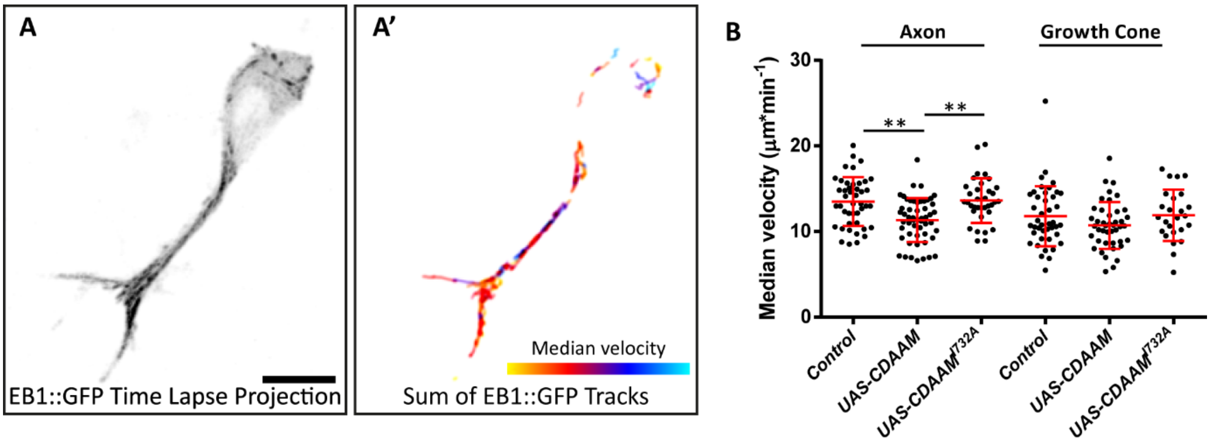

Supplementary Figure S4. Live imaging analysis of microtubule growth in control and CDAAM expressing primary neurons.
(A) Representative time lapse projection of a primary neuron expressing EB1::GFP to detect the growing (+) end of microtubules. (A`) Time lapse projection of detected EB1::GFP tracks, color coded according to their median velocity (from yellow to cyan the values are between 3,54-29,52 µm/min). (B) Scatterplots show the median velocity of EB1::GFP tracks derived from control (*Elav-Gal4; UAS-EB1::GFP*) and CDAAM or CDAAM^{I732A} expressing embryos. Microtubule growth velocity was evaluated in the growth cone and axon separately. Kruskal-Wallis test was used for statistical analysis. Dunn`s *post-hoc* test was used for multiple comparison. ** $p < 0.01$. Scale bar represents 5 µm.

Primer name	Sequence
DAAM-FH2-mutant-1Fw	gaagcttacgcagggcagcggaagcagccacctcacgagaggcctc
DAAM-FH2-mutant-1Rev	gaggcctctcgtgaggtggctgcttccgctgccctgcgtaagcttc
DAAM-FH2-mutant-2Fwd	ctgcttccgctgccctggctgcgcttctggagttggtcc
DAAM-FH2-mutant-2Rev	ggaccaactccagaagcgcagccagggcagcggaagcag

Supplementary Table S1. List of primers used for generation of the FH2$^{R/K\text{-}A}$ mutant.
Due to the high number of nucleotide changes, the mutagenesis was performed in two separate PCR reactions.
